# Functionalized Magnetic Nanoparticles: Can They Revolutionize the Treatment of Neurodegenerative Disorders?

**DOI:** 10.3390/ma18184302

**Published:** 2025-09-14

**Authors:** Nikolay Zahariev, Radka Boyuklieva, Dimitar Penkov, Paolina Lukova, Plamen Katsarov

**Affiliations:** 1 Department of Pharmaceutical Technology and Biopharmacy, Faculty of Pharmacy, Medical University of Plovdiv, 4002 Plovdiv, Bulgaria; nikolay.zahariev@mu-plovdiv.bg (N.Z.); radka.boyuklieva@mu-plovdiv.bg (R.B.); dimitar.penkov@mu-plovdiv.bg (D.P.); 2 Research Institute at Medical University of Plovdiv (RIMU), 4002 Plovdiv, Bulgaria; 3 Department of Pharmacognosy and Pharmaceutical Chemistry, Faculty of Pharmacy, Medical University of Plovdiv, Vasil Aprilov Str. 15A, 4002 Plovdiv, Bulgaria; paolina.lukova@mu-plovdiv.bg

**Keywords:** magnetic nanoparticles, neurodegenerative disorders, diagnostics and therapy

## Abstract

Neurodegenerative disorders (NDs), including Alzheimer’s disease and Parkinson’s disease, pose a significant global health challenge characterized by progressive neuronal loss and limited therapeutic options. Early diagnosis remains a considerable hurdle due to the absence of reliable biomarkers and the restrictive nature of the blood–brain barrier (BBB), which complicates effective drug delivery. Magnetic nanoparticles (MNPs), particularly those based on iron oxide, have emerged as promising tools for both diagnostic and therapeutic applications in NDs, thanks to their superparamagnetism, biocompatibility, and customizable surfaces. This review examines various synthesis strategies for MNPs, encompassing physical methods (such as lithography, ball milling, and laser ablation) and chemical approaches (co-precipitation, thermal decomposition, hydrothermal synthesis, sol–gel processes, and polyacrylamide gel techniques), while highlighting how these techniques influence particle properties. This review also explores recent advancements in surface functionalization using polymers and coatings to enhance circulation time in the bloodstream and improve BBB penetration for targeted delivery. Furthermore, it emphasizes both in vitro and in vivo applications, showcasing MNPs’ effectiveness in enhancing imaging sensitivity and enabling targeted drug and gene delivery. By linking synthesis methods, functionalization techniques, and biomedical outcomes, this review illustrates the transformative potential of MNPs as next-generation theranostic agents in precision medicine for neurodegenerative diseases.

## 1. Introduction

Neurodegenerative disorders (NDs) pose a significant challenge to global public health and represent a growing social burden, increasingly evident as life expectancy rises [1]. Conditions such as Alzheimer’s disease (AD), Parkinson’s disease (PD), Huntington’s disease (HD), and amyotrophic lateral sclerosis (ALS) affect millions of individuals worldwide, with their prevalence expected to increase due to population aging [2]. These disorders are associated with diverse pathological mechanisms and are characterized by the progressive loss of neuronal structure or function. Common underlying features include abnormal protein aggregation, neuroinflammation, oxidative stress, and dysregulation of neurotransmitter systems [3,4]. Despite extensive research efforts, current diagnostic approaches remain suboptimal, relying primarily on clinical evaluations, neuropsychological assessments, and neuroimaging techniques that often detect the disease only after substantial and irreversible neuronal damage has occurred [5]. Similarly, most available treatments are palliative, focused on symptom management rather than modifying disease progression [5,6,7]. This critical gap underscores the need for innovative solutions capable of addressing both diagnostic and therapeutic shortcomings. Functionalized nanomaterials—and magnetic nanoparticles (MNPs) in particular—are emerging as leading candidates because they can integrate early biomarker detection [8,9,10,11,12,13], targeted drug delivery [14], and non-invasive monitoring into a single technological platform [15,16].

The etiology of AD is not yet fully elucidated, though it is widely believed to result from a multifactorial interplay of genetic predispositions and environmental influences [7]. AD is marked by the accumulation of amyloid-beta plaques and neurofibrillary tangles, leading to progressive neuronal loss. Early symptoms often involve memory impairment and difficulty acquiring new information. As the disease advances, patients experience severe cognitive decline, communication deficits, and loss of independence in daily activities. Globally, AD accounts for an estimated 60–70% of dementia cases [1,2]. Advancing age is the most prominent risk factor, with the majority of diagnoses occurring in individuals aged 65 years and older [17]. Recent studies also emphasize the importance of early detection, particularly at the prodromal or mild cognitive impairment stage, where therapeutic interventions may be more effective [18,19,20]. Functionalized MNPs designed to bind amyloid-beta, tau, or associated biomarkers in peripheral biofluids or brain tissue represent an emerging strategy to achieve such early-stage diagnosis while simultaneously enabling image-guided therapy [21,22].

PD is the second most common, chronic, progressive ND after AD, affecting over 10 million people worldwide [6]. It is characterized by the selective degeneration of dopaminergic neurons in the substantia nigra, resulting in dopamine depletion in the striatum. This leads to classic motor symptoms such as resting tremors, rigidity, and bradykinesia. In addition to motor dysfunction, PD is frequently associated with non-motor symptoms, including mood disturbances, cognitive impairment, and sleep disorders [6]. Age is a key risk factor, with incidence rising sharply after the age of 60 and peaking in individuals over 80. Epidemiological studies also suggest a higher prevalence of PD in males, potentially due to hormonal or behavioral factors [23]. Current research has increasingly focused on the pathogenic role of alpha-synuclein aggregation, oxidative stress, and neuroinflammation, all of which provide promising molecular targets for intervention [3,24]. In this context, functionalized MNPs can be engineered for dual applications: (i) to detect and monitor pathological protein aggregates using MRI or liquid-biopsy–based assays [25,26,27,28,29], and (ii) to deliver neuroprotective or gene-silencing agents directly to the affected brain regions, guided by external magnetic fields [30].

One of the major challenges in the diagnosis and treatment of NDs lies in the complexity of the central nervous system (CNS) and the protective nature of the blood–brain barrier (BBB) [31]. This highly selective physiological barrier limits the entry of most pharmacological agents and imaging molecules into the brain parenchyma. As a result, effective drug delivery and early visualization of pathological changes remain significant hurdles for conventional therapeutic and diagnostic strategies [32]. In response to these challenges, nanoparticle (NP)-based delivery systems, particularly MNPs, have emerged as a promising technological platform. Their unique physicochemical properties and functional adaptability enable targeted drug delivery and enhanced imaging capabilities [32]. Functionalization strategies, including polymer coatings (e.g., dextran, chitosan, PEG), lipid-based assemblies, and bioactive ligand conjugation, have been shown to prolong circulation time, improve BBB penetration, and enable receptor-mediated transport [33,34,35,36,37]. These modifications not only enhance delivery efficiency but also open opportunities for precision medicine approaches that tailor nanoparticle design to specific disease mechanisms [38].

NP-based therapeutic strategies have gained substantial momentum in recent years as a means of overcoming the limitations of conventional treatments for NDs [39]. Their nanoscale dimensions enable improved pharmacokinetics, cellular uptake, and, crucially, penetration across the BBB [40]. Among these, MNPs offer distinct advantages due to their external field-guided navigation, multifunctional surface modification, and theranostic potential—allowing simultaneous drug delivery and real-time imaging [41]. MNPs can be functionalized with targeting ligands, enzymes, neuroprotective agents, or gene-silencing molecules such as siRNA or antisense oligonucleotides [42,43]. This facilitates highly specific interactions with pathological sites, such as amyloid plaques in AD or alpha-synuclein aggregates in PD [7]. Furthermore, MNPs can be engineered for controlled, on-demand drug release, triggered by external magnetic fields or environmental cues (e.g., pH, temperature). Several preclinical studies have demonstrated that MNPs loaded with antioxidants, anti-inflammatory drugs, or neurotrophic factors can reduce oxidative stress, suppress neuroinflammation, and enhance neuronal survival in models of AD and PD [44]. As research advances, MNPs are expected to play an increasingly central role in multifunctional nanomedicine platforms that unify targeted therapy, imaging, and biomarker monitoring, offering a path toward personalized and disease-modifying interventions in neurodegenerative care [39,40].

This review examines the primary physical and chemical methods used in the synthesis of MNPs and investigates how these approaches affect particle morphology and size. It further highlights recent advances in both in vivo and in vitro biomedical applications of surface-modified MNPs, with a specific focus on brain-targeted imaging and the delivery of neurotherapeutics. Special attention is given to their application in the diagnosis and treatment of AD and PD. In contrast to earlier reviews that have often discussed synthesis methods, surface functionalization, or biomedical applications in isolation, the present work aims to provide a holistic perspective by linking these domains into a unified framework. Specifically, we emphasize how advances in nanoparticle engineering translate into functional outcomes such as BBB penetration, biomarker detection, and disease-modifying efficacy in AD and PD. By synthesizing these developments, this review underscores the innovative potential of MNPs as next-generation theranostic agents for NDs.

## 2. Neurodegeneration Mechanism and Types of Neurodegenerative Disorders

**Figure 1:**
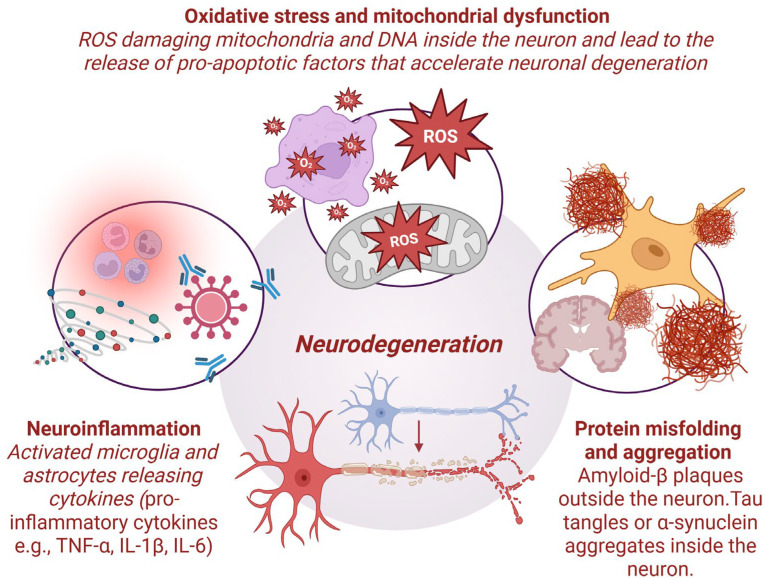
Pathological mechanisms in neurodegeneration (created in https://BioRender.com).

Neurodegeneration is a complex pathological process characterized by a decline in the functions of specific nerve cells in the brain. While often associated with aging, NDs can also result from genetic mutations, environmental factors, or a combination of both. Common conditions within this category include AD, PD, HD, and ALS. Despite clinical heterogeneity, these disorders share several key pathogenic mechanisms, including oxidative stress, mitochondrial dysfunction, protein misfolding, and neuroinflammation [45]. Advances in immunohistochemistry have enabled the identification of disease-specific protein aggregates and their propagation patterns throughout the nervous system [46]. Notably, these misfolded proteins are not restricted to the CNS; they are also detectable in peripheral tissues and biofluids, emphasizing the complexity of ND diagnosis and the critical role of reliable biomarkers and neuroimaging techniques [47]. The following sections discuss the major molecular mechanisms implicated in neurodegeneration (Figure 1).

### 2.1. Oxidative Stress and Mitochondrial Dysfunction

Oxidative stress plays a central role in neuronal injury, arising from an imbalance between the generation of reactive oxygen species (ROS) and reactive nitrogen species (RNS) and the cell’s antioxidant defenses. This imbalance activates pro-inflammatory pathways and damages essential biomolecules, including DNA, proteins, and lipids, thereby accelerating cellular aging [48]. S-nitrosylation—a reaction that modifies protein thiol groups—can alter nitric oxide bioactivity, exerting either neuroprotective or neurotoxic effects, depending on the target protein. In AD, elevated nitrosative stress impairs mitochondrial respiration, disrupts synaptic function, and promotes neuronal apoptosis [49]. Additionally, amyloid-beta accumulation and aging exacerbate oxidative stress, contributing to cerebrovascular dysfunction [48]. While antioxidant therapies have shown promise in alleviating cognitive decline and inflammation, chronic oxidative stress remains closely associated with increased inflammatory cytokine levels, which are linked to cognitive deficits in elderly individuals [50].

The brain, which demands approximately 20% of the total body’s oxygen, relies primarily on mitochondrial oxidative phosphorylation for energy production. This process generates adenosine triphosphate (ATP), which fuels essential activities such as neurotransmission and ion homeostasis via Na^+^/K^+^-ATPase [51]. Mitochondria also regulate calcium signaling, apoptosis, and cellular metabolism, maintaining integrity through dynamic processes such as fission, fusion, and mitophagy. Dysregulation of these processes leads to mitochondrial dysfunction, characterized by impaired ATP synthesis and excessive ROS production [52]. Oxidative damage to mitochondrial DNA, lipids, and proteins creates a self-perpetuating cycle of injury. Furthermore, mitochondrial components released into the cytoplasm or extracellular milieu can activate innate immune pathways, exacerbating neuroinflammation. Evidence suggests that mitochondrial dysfunction appears early in many NDs, serving as both a driver and a consequence of neurodegeneration [53,54].

### 2.2. Protein Misfolding and Aggregation

Protein misfolding and aggregation are pathological hallmarks of various NDs. Under normal conditions, molecular chaperones and the ubiquitin-proteasome system maintain proteostasis by ensuring the proper folding and degradation of proteins. However, under stress or pathological conditions, misfolded proteins may evade these quality control systems, accumulating as toxic aggregates [55]. These aggregates often originate from misfolded protein “seeds” that induce further misfolding and aggregation of native proteins. In AD, these processes result in amyloid-beta plaques and tau neurofibrillary tangles; in PD, alpha-synuclein aggregates form Lewy bodies; in HD, mutant huntingtin proteins with polyglutamine expansions accumulate; and in ALS and frontotemporal dementia, TAR DNA-binding protein 43 (TDP-43) aggregates are involved [56,57]. These protein inclusions disrupt cellular homeostasis through mechanisms such as oxidative stress induction, mitochondrial impairment, synaptic dysfunction, and inflammatory activation. Elucidating the molecular pathways governing protein aggregation offers promising therapeutic targets.

### 2.3. Neuroinflammation

Neuroinflammation involves the activation of glial cells, primarily microglia and astrocytes, within the CNS. While transient inflammation serves a protective role, chronic neuroinflammation contributes significantly to neuronal damage and disease progression [58]. Microglia, the brain’s resident immune cells, play a crucial role in clearing debris and misfolded proteins, such as amyloid-beta. However, in pathological states, they can become persistently activated, releasing pro-inflammatory cytokines and reactive species that damage surrounding neurons [17,59]. Astrocytes, which normally support neuronal function and maintain the BBB, also undergo reactive changes in disease, leading to altered gene expression, cellular hypertrophy, and loss of supportive roles [60]. A central regulator of these inflammatory processes is the nuclear factor-kappa B (NF-κB) signaling pathway. Its activation in glial cells induces the production of inflammatory mediators that perpetuate neurotoxicity [61,62]. Sustained activation of NF-κB has been observed across multiple NDs, including AD, PD, HD, and ALS, underscoring its potential as a therapeutic target [24].

## 3. Magnetic Nanoparticles: Fundamentals and Biomedical Potential

**Figure 2:**
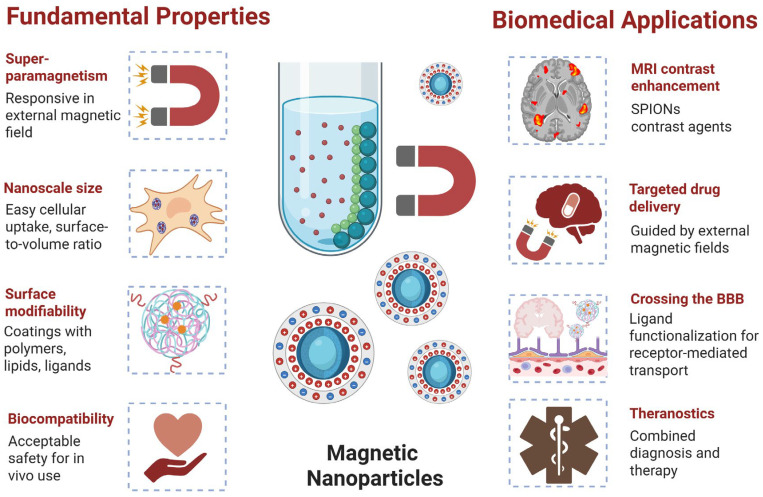
Fundamental properties and biomedical potential of magnetic nanoparticles (created in https://BioRender.com).

MNPs, especially those composed of iron oxides such as magnetite (Fe_3_O_4_) and maghemite (γ-Fe_2_O_3_), have attracted considerable attention in biomedical research due to their unique magnetic properties, nanoscale dimensions, and surface modifiability (Figure 2). Their most notable characteristic—superparamagnetism—enables these particles to be magnetized only in the presence of an external magnetic field and lose their magnetization upon removal of the field. This prevents residual magnetization and aggregation, making MNPs particularly suitable for in vivo biomedical applications [63]. One of the most prominent uses of MNPs is in diagnostic imaging, especially in magnetic resonance imaging (MRI). Superparamagnetic iron oxide nanoparticles (SPIONs), a subclass of MNPs, act as negative contrast agents in T_2_-weighted MRI scans, significantly enhancing imaging sensitivity and resolution. Compared to conventional gadolinium-based contrast agents, SPIONs exhibit lower toxicity, longer blood circulation times, and improved safety profiles, which are significant for patients with renal insufficiency or other comorbidities [63].

Beyond imaging, MNPs play a central role in therapeutic delivery. Their small size and modifiable surfaces allow for the conjugation or encapsulation of drugs, nucleic acids, or biomolecules. These particles can be guided using external magnetic fields to specific anatomical regions, such as damaged brain tissues, providing a means for targeted drug delivery. Moreover, MNPs can be engineered to respond to various stimuli, including pH changes, enzymatic activity, or magnetic fields, to achieve controlled and site-specific release of their therapeutic cargo, thus reducing off-target effects and enhancing treatment efficacy [44,64,65]. A particularly valuable application of MNPs in neuroscience is their ability to cross the BBB. The BBB is a selective barrier that limits the entry of most systemic drugs into the CNS, posing a significant obstacle in treating NDs. Functionalization of MNPs with targeting ligands such as transferrin, antibodies, or peptides facilitates receptor-mediated transcytosis across the BBB [32]. In parallel, magnetic targeting can further guide NPs to specific brain regions, enhancing local accumulation and therapeutic impact [44].

In addition to delivery and imaging, MNPs are being explored in liquid biopsy platforms for non-invasive diagnostics. These NPs can be functionalized to capture circulating biomarkers such as amyloid-beta, tau, microRNAs, and extracellular vesicles (EVs) from blood samples [66]. For instance, transferrin-coated MNPs have been used to isolate brain-derived EVs containing diagnostic microRNAs in PD, while antifouling SPIONs have successfully detected amyloid-beta and tau in the peripheral blood of AD patients [23]. The theranostic potential of MNPs—their combined diagnostic and therapeutic capabilities—represents a significant advancement in personalized medicine. A single MNP system can serve as an MRI contrast agent, a vehicle for targeted therapy, and a biosensor for disease monitoring. Such multifunctionality offers real-time assessment of drug delivery, disease progression, and therapeutic efficacy in a minimally invasive manner [66].

Collectively, the unique combination of magnetic responsiveness, functional adaptability, and biocompatibility positions MNPs as a powerful tool in the diagnosis and treatment of NDs. As research progresses, further innovations in MNP engineering, coating strategies, and biological targeting will continue to expand their impact in clinical neuroscience.

## 4. Methods for Preparing Magnetic Nanoparticles

In recent years, significant advancements have been made in the development of methods for synthesizing MNPs. The choice of method depends on the desired particle size, morphology, magnetic properties, and stability [67]. Although other strongly magnetic nanoparticles, such as BaFe_12_O_19_ and SrFe_12_O_19_, have been synthesized, their limited biocompatibility restricts their biomedical use. Therefore, this review focuses on iron oxide–based nanoparticles (e.g., magnetite, maghemite), which represent the most clinically relevant systems for diagnosis and therapy [68,69]. These techniques can be broadly categorized into physical and chemical approaches (Table 1) [67].

### 4.1. Physical Methods

**Figure 3:**
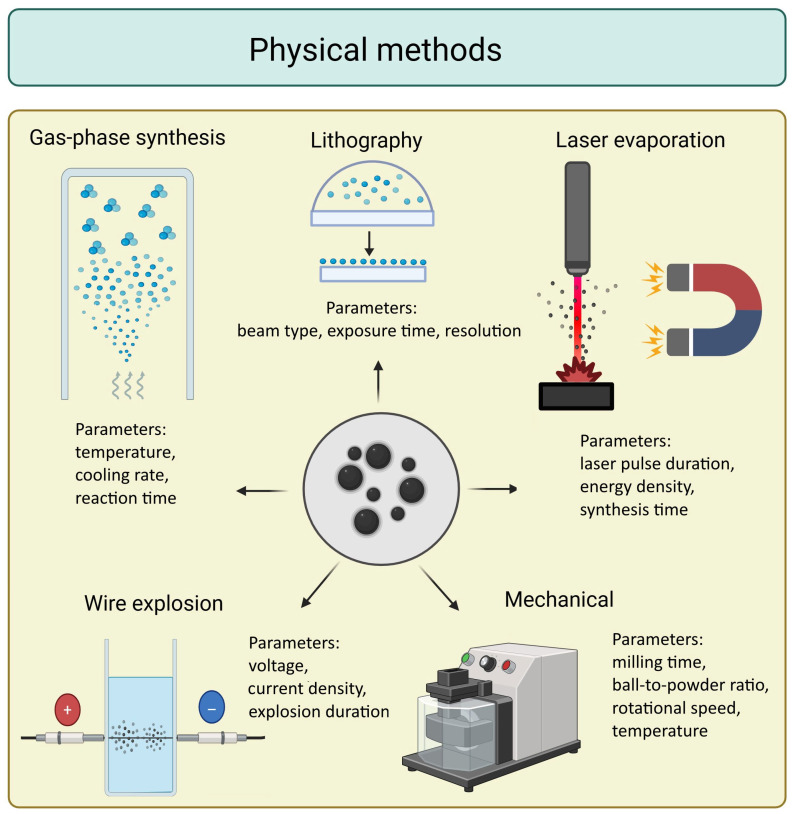
Physical methods for the preparation of magnetic nanoparticles and some of their key process parameters (created in https://BioRender.com).

Physical methods (Figure 3) involve mechanical or physical processes to produce NPs either from bulk materials (top-down) or by assembling atoms or molecules (bottom-up) [67].

One notable physical technique is **lithography**, a precise patterning method that offers control over NP shape, size, and spatial distribution [67]. This technique is generally comparable to a printing process, where light or electron beams are used to shape a material into the desired nanostructured arrays from a suitable precursor. Advanced forms such as scanning nanosphere lithography, colloidal lithography, and soft nanoimprinting have enhanced the applicability of lithography in biomedical and electronic fields [70].

**Ball milling**, a top-down method introduced in the 1970s, uses mechanical grinding to reduce coarse particles to the nanoscale. It is cost-effective and straightforward, but has drawbacks, including contamination from the milling medium and broad particle size distributions [71].

Among bottom-up methods, **laser ablation** involves irradiating a solid target with high-energy laser pulses, resulting in localized vaporization. The vapor subsequently condenses into NPs in a gaseous medium. This technique offers excellent control over purity and composition but requires complex instrumentation [72].

**Wire explosion**, another emerging method, utilizes high-voltage electrical currents to explode thin metal wires, generating NPs. This approach is clean, safe, and environmentally friendly, but suffers from poor control over particle size distribution, limiting its utility in precision-demanding applications [73].

**Gas-phase synthesis** is another bottom-up approach that generates NPs directly from gaseous precursors [74]. The two main types of this method include **chemical vapor condensation**, where high-temperature reduction in metal vapors forms NPs, and **inert gas condensation**, which involves cooling vaporized metals in an inert atmosphere to promote nucleation. These techniques enable excellent control over particle composition and structure, although they often require specialized equipment and high-temperature-resistant metals [75].

### 4.2. Chemical Methods

**Figure 4:**
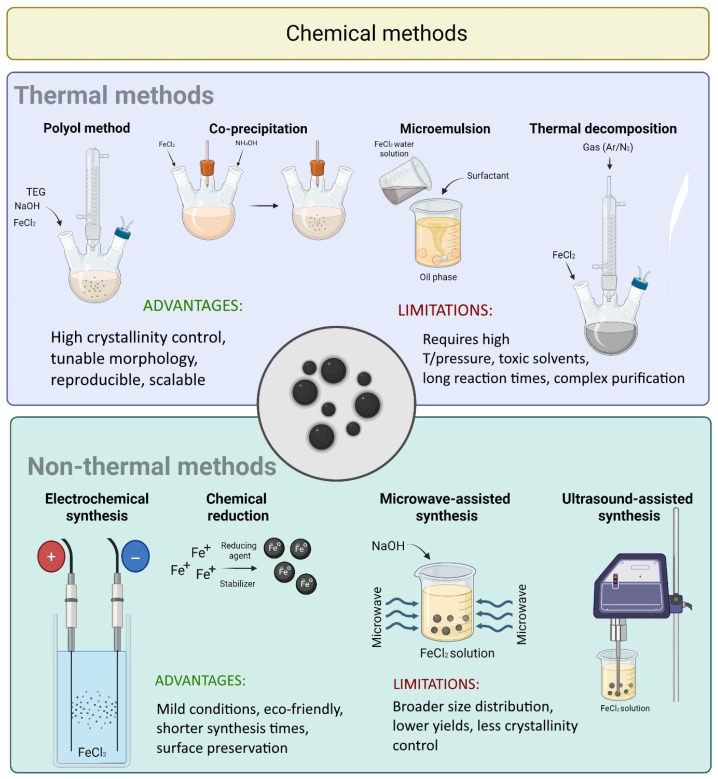
Chemical methods for the preparation of magnetic nanoparticles (created in https://BioRender.com).

Chemical synthesis methods (Figure 4) are among the most widely employed for MNPs preparation due to their scalability, reproducibility, and ability to fine-tune particle characteristics [67].

**Co-precipitation** is one of the most common and straightforward chemical techniques. It involves the simultaneous precipitation of ferrous (Fe^2+^) and ferric (Fe^3+^) ions in an alkaline medium to form magnetite (Fe_3_O_4_) or maghemite (γ-Fe_2_O_3_) NPs [76]. This method is cost-effective and allows control over particle size and magnetic properties by adjusting parameters such as pH, temperature, and ionic strength [77,78]. For example, manganese ferrite (MnFe_2_O_4_) NPs can be synthesized using ferric chloride (FeCl_3_) and manganese chloride (MnCl_2_) in the presence of sodium hydroxide as the precipitating agent [79].

To address the main drawbacks of co-precipitation, the **thermal decomposition** method was developed. It yields highly crystalline and uniformly sized MNPs by decomposing organometallic compounds at elevated temperatures in organic solvents, often in the presence of surfactants [77,80]. Common precursors include acetylacetonates, N-nitrosophenylhydroxylamines, and metal carbonyls (Fe^2+/3+^, Mn^2+/3+^, Co^2+/3+^, Ni^2+/3+^), while stabilizers like fatty acids, oleic acid, and hexadecylamine are frequently used [81]. Reaction parameters—such as temperature, time, solvent type, and surfactant—strongly influence the resulting NP size and morphology [82].

Jana et al. [83] demonstrated that iron oxide MNPs synthesized from metal fatty acid salts formed stable, monodispersed particles ranging from 6 to 50 nm. Increasing the reaction time led to a morphological transition from spherical to cubic particles. Despite its advantages, this method involves toxic organic solvents and typically requires extensive purification [84].

The **hydrothermal method** utilizes elevated pressure and temperature in sealed reactors to form NPs through nucleation and controlled growth [84,85]. This process involves hydrolysis and oxidation reactions, with particle morphology and crystallinity influenced by pressure, temperature, solvent, and reaction duration [86,87]. Li et al. synthesized spherical Fe_3_O_4_ NPs (15 nm) using this method for MRI-based tumor imaging [88], while another study reported chitosan-coated Fe_3_O_4_ NPs (25 nm) suitable for enzyme immobilization [89].

**The polyol method** uses polyols as solvents, reducing agents, and complexing agents, enabling the synthesis of MNPs with various morphologies, including single-core, multicore, hollow spheres, and nanoflowers [77,84]. Reaction temperature can be used to control particle size [90]. This method is regarded as cost-effective, environmentally friendly, and suitable for industrial applications [91].

The **sol–gel method** involves the hydrolysis and polycondensation of metal alkoxides to form a homogeneous sol, which subsequently transitions into a gel [85,92]. After solvent evaporation and thermal treatment, the resulting gel forms MNPs [93].

**Microemulsion techniques** rely on the formation of thermodynamically stable mixtures of water and oil phases using surfactants. Direct (oil-in-water) and reverse (water-in-oil) emulsions allow fine control over particle size, shape, and composition, though limitations include low yield and high solvent consumption [76].

Additionally, several **non-thermal chemical techniques** are gaining interest [94]. **Chemical reduction techniques** use mild reducing agents at ambient conditions, minimizing thermal stress and preserving delicate surface features [95]. **Electrochemical synthesis** employs electric potentials to precisely regulate particle size and morphology [96].

**Microwave-assisted synthesis** uses electromagnetic radiation for rapid and uniform heating, shortening reaction times and increasing yields. This method has been successfully applied to various MNP systems [97]. **Ultrasound-assisted synthesis** enhances mixing and nucleation through acoustic cavitation, improving reaction efficiency and promoting size uniformity [98].

In addition, the **polyacrylamide gel method** has been reported as an efficient and versatile route for preparing ferrite-based MNPs [99]. In this technique, polyacrylamide serves as a gelling and stabilizing agent, creating a uniform polymeric network that entraps metal precursors and promotes controlled nucleation during subsequent thermal treatment. This confinement effect minimizes particle agglomeration and allows fine-tuning of particle size, morphology, and crystallinity. For example, Hassanzadeh-Tabrizi et al. synthesized NiFe_2_O_4_ nanoparticles with an average size of 90 nm using the polyacrylamide gel route, achieving well-defined crystalline structures and desirable magnetic properties, including distinct saturation magnetization and coercivity [100]. More recently, Ling et al. demonstrated the successful preparation of Zn-doped CoFe_2_O_4_ nanoparticles using a modified polyacrylamide gel approach, showing that dopant concentration could precisely modulate the crystallite structure and magnetic response [101]. Beyond spinel ferrites, this method has also been applied to other ferrite systems such as MgFe_2_O_4_, CaFe_2_O_4_, BaFe_2_O_4_, and (Ba, Sr) Fe_12_O_19_, underscoring its broad applicability in tailoring physicochemical and magnetic properties for biomedical use [102]. Compared to widely used chemical routes such as co-precipitation, which often yields broader size distributions, or sol–gel processing, which requires multiple steps and higher calcination temperatures [77,78], the polyacrylamide gel method offers a relatively simple, cost-effective, and reproducible approach with excellent control over particle homogeneity and crystallinity. The scalability and tunability of this synthesis route make it a promising alternative for fabricating high-quality MNPs designed for diagnostic and therapeutic applications [99].

Overall, the synthesis of MNPs can be achieved through a wide range of physical and chemical methods, each with distinct benefits and limitations. Physical techniques such as lithography and laser ablation offer excellent control over purity and particle architecture but remain constrained by scalability and cost [67,70,72]. By contrast, chemical approaches—including co-precipitation, hydrothermal synthesis, polyol and sol–gel routes—are widely adopted because of their reproducibility, tunability, and suitability for large-scale production, though challenges such as particle aggregation, the use of toxic solvents, and purification requirements persist [76,77,91]. Among these, the thermal decomposition, hydrothermal, and polyacrylamide gel methods stand out as particularly promising due to their ability to produce highly crystalline, monodisperse particles with controllable morphology and tailored magnetic properties [82,86]. Importantly, the polyacrylamide gel method provides a versatile and cost-effective alternative with strong potential for biomedical translation, as it combines scalability with precise control over crystallinity and particle homogeneity [99,102]. Taken together, these comparative insights suggest that future research should focus on hybridizing chemical routes, optimizing environmentally friendly processes, and developing standardized protocols that balance scalability with biomedical applicability. This provides a strong foundation for the subsequent discussion on surface functionalization strategies that further enhance the utility of MNPs in medical applications.

**Table 1:** Comparative overview of magnetic nanoparticle (MNP) synthesis methods.

Method	Key Features	Pros	Cons	Comments	Ref.
Physical Methods					
Lithography	Patterning using light/electron beams; top-down precision	High control over size/shape; reproducible nanostructures	Expensive, low throughput, limited scalability	Useful for biosensors and electronic applications, less common in biomedicine	[67]
Ball milling	Mechanical grinding of bulk precursors	Simple, low cost, scalable	Broad size distribution; contamination from milling media	Better suited for large-scale powder production, less for biomedical quality	[71]
Laser ablation	High-energy laser irradiation of bulk target; bottom-up route	High purity; excellent control over composition	Requires complex, costly instrumentation; limited scalability	Suitable for research-grade materials, less practical for routine synthesis	[72]
Wire explosion	NP generation from exploding metal wires under high voltage	Clean, eco-friendly; no need for solvents	Poor size control; irregular morphology	Attractive for rapid synthesis but lacks uniformity for biomedical use	[73]
Gas-phase methods	Vapor condensation under inert/reducing gases	Good structural control; high-purity NPs	Requires high temperatures; specialized equipment	Promising for metal-based NPs, less common in bioapplications	[74]
	Chemical Methods				
Co-precipitation	Simultaneous precipitation of Fe^2+^/Fe^3+^ in alkaline medium	Easy, cost-effective, scalable, adjustable parameters	Tends to produce aggregation; broad size distribution	Widely used for biomedical Fe_3_O_4_ NPs	[76]
Thermal decomposition	High-T decomposition of organometallic precursors in organic solvents	Monodisperse, highly crystalline NPs with tunable size/morphology	Toxic solvents; expensive precursors; purification required	Suitable for precision applications (MRI contrast, drug delivery)	[77]
Hydrothermal	High-T, high-P reactions in sealed reactors	Produces uniform particles with good crystallinity; can tune morphology	Requires autoclaves; longer reaction times	Used for Fe_3_O_4_, doped ferrites, coating integration	[84]
Polyol method	Polyols act as solvent, reducing and stabilizing agents	Eco-friendly, scalable; versatile morphologies (spheres, flowers, hollow)	Reaction conditions can be complex; requires careful control	Industrially promising and environmentally safer	[77]
Sol–gel	Hydrolysis and polycondensation of metal alkoxides	Fine control over structure; homogeneous products	Multi-step; high calcination T; risk of particle growth	Good for complex oxides, less for biomedical-grade MNPs	[92]
Microemulsion	Thermodynamically stable oil–water–surfactant mixtures	Precise control over particle size/shape	Low yield; large solvent use; difficult scale-up	Useful for research-scale synthesis of uniform NPs	[76]
Chemical reduction	Mild reducing agents for NP formation at ambient conditions	Simple; preserves surface features	Limited to certain metal salts; stability issues	Useful for biocompatible coatings	[95]
Electrochemical	Electrolysis-based control of NP formation	Precise size/morphology control	Requires specialized equipment; low yield	Good for experimental fine-tuning	[96]
Microwave-assisted	Uniform, rapid heating with microwaves	Fast, energy-efficient; higher yields	Limited penetration depth; uneven heating in bulk	Scalable for small-batch synthesis	[97]
Ultrasound-assisted	Acoustic cavitation promotes nucleation and mixing	Improves reaction kinetics, particle uniformity	Equipment cost; potential for uncontrolled local heating	Useful as a supporting/combined method	[98]
Polyacrylamide gel	Metal precursors embedded in polymer gel, thermally treated	Monodisperse, crystalline; reproducible; scalable; cost-effective	Requires calcination; some risk of organic residue	Promising alternative for ferrites (MgFe_2_O_4_, CaFe_2_O_4_, BaFe_2_O_4_, Ba/SrFe_12_O_19_)	[99]

## 5. Surface Functionalization and Polymer Coatings

**Figure 5:**
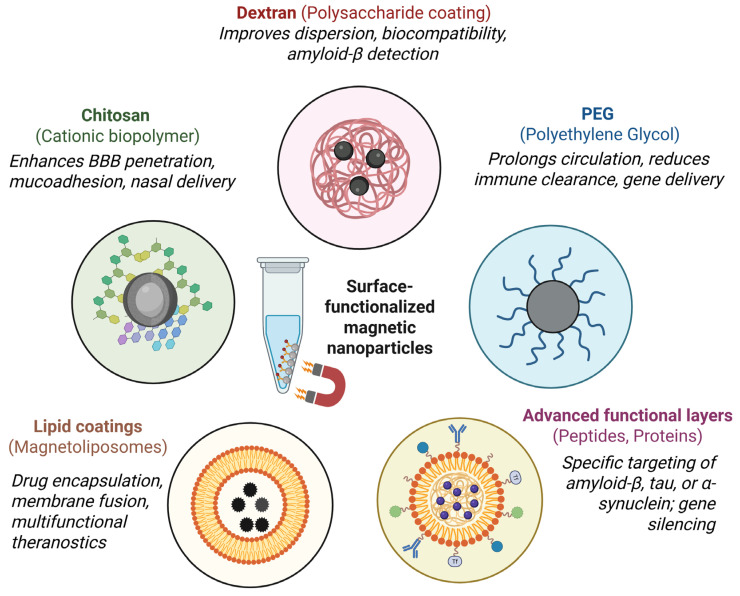
Surface functionalization strategies for magnetic nanoparticles in neurodegenerative disorders (created in https://BioRender.com).

Surface functionalization of MNPs with biocompatible polymers plays a pivotal role in enhancing their physical stability, circulation time, biocompatibility, and targeting ability. Coatings prevent core oxidation and aggregation, enable drug and gene loading, allow ligand attachment, and support controlled release mechanisms (Table 2). The choice of polymer depends on the intended biomedical application: hydrophilic polymers (e.g., dextran, polyethylene glycol) improve colloidal stability and reduce immune recognition, chitosan enhances mucoadhesion and BBB penetration, while lipid-based layers support drug encapsulation and membrane fusion. These modifications are essential for enabling MNPs to overcome biological barriers, particularly the BBB, in the treatment and diagnosis of NDs (Figure 5) [103].

### 5.1. Polymer-Based Coatings

#### 5.1.1. Dextran

Dextran, a non-toxic, hydrophilic polysaccharide, is widely used as a coating agent for SPIONs. It improves particle dispersion in aqueous solutions and stability under physiological conditions. It is often selected because it improves aqueous dispersibility, provides biocompatibility, and reduces aggregation in physiological conditions. Early dextran-coated SPIONs developed by Molday et al. exhibited 15 nm magnetic cores with overall sizes between 30 and 40 nm and demonstrated efficient cell targeting capabilities [104]. Later studies, such as that by Katebi et al., showed that dextran sulfate-coated SPIONs loaded with quercetin exhibited significantly lower cytotoxicity than dimercaptosuccinic acid-coated SPIONs [105].

Dextran coatings have also enabled the detection and inhibition of amyloid-beta in AD. For example, Kouyoumdjian et al. developed dextran-coated glycol-NPs conjugated with ganglioside-mimicking ligands that selectively bound amyloid-beta 1–42, reduced its cytotoxicity in SH-SY5Y cells, and were detectable by MRI and Prussian blue staining [106]. In PD, dextran-coated SPIONs enhanced the migration and therapeutic efficacy of human mesenchymal stem cells toward damaged dopaminergic neurons, suggesting their utility in stem cell-based PD therapies [36].

#### 5.1.2. Chitosan

Chitosan, another biopolymer derived from chitin, is positively charged and biodegradable, with mucoadhesive properties that enhance drug transport across epithelial barriers [33,107]. Its cationic nature and mucoadhesion make it especially useful for enhancing BBB penetration and nasal delivery routes. In comparative studies, chitosan NPs and chitosan-coated MNPs both loaded with tacrine improved cognitive behavior in AD rat models. The coated MNPs provided better BBB penetration and increased the expression of the neuroprotective gene Seladin-1 [34,108].

#### 5.1.3. Polyethylene Glycol

Polyethylene glycol (PEG) is a hydrophilic polymer known for reducing NP aggregation, prolonging circulation, and minimizing immune system recognition [109]. PEG is often chosen for systemic administration because it confers “stealth” properties, extends half-life, and reduces opsonization and clearance by the reticuloendothelial system. PEG-coated SPIONs synthesized through anionic ring-opening polymerization showed enhanced stability and reduced uptake by macrophages [110]. Mirsadeghi et al. demonstrated that the surface charge of PEG-coated SPIONs influenced their impact on amyloid-beta fibrillation—positively charged particles could inhibit or promote fibril formation depending on the concentration, while neutral and negatively charged particles consistently suppressed aggregation [37].

López-Barbosa et al. created PEGylated SPIONs for intracellular siRNA delivery targeting the BACE1 gene, a key player in amyloid-beta generation. These NPs enhanced gene silencing efficiency in fibroblasts [42]. In another study, Li et al. synthesized PEG-block-allyl glycidyl ether-coated SPIONs conjugated with antibodies for the detection of both amyloid-beta and tau proteins in blood samples. These particles outperformed commercial Dynabeads^®^ in sensitivity and specificity [111].

#### 5.1.4. Lipid Coatings

Lipid-coated MNPs, or magnetoliposomes, combine the stability and magnetic responsiveness of SPIONs with lipid biocompatibility and membrane fusion capabilities. They are selected when drug encapsulation, membrane fusion, or multifunctional theranostics are required. This hybrid system offers a powerful platform for theranostics—simultaneous diagnosis and therapy [112]. Hu et al. designed DSPE-PEG NPs loaded with Congo red and Rutin, which selectively bound amyloid-beta, reduced aggregation, and exhibited antioxidant properties [113]. Similarly, curcumin-loaded SPIONs encapsulated by DSPE-PEG showed specific binding to transferrin receptors and early amyloid-beta plaques, effectively inhibiting aggregation by suppressing NLRP3 inflammasome activity [114].

Oleic acid is another commonly used surface modifier that provides hydrophobic stabilization and promotes interactions with lipid membranes. Its hydrophobicity makes it suitable for stabilizing NPs in organic phases and for enhancing lipid–NP interactions. Zablotskaya et al. synthesized oleic acid-coated iron oxide NPs (~11 nm), which exhibited size- and condition-dependent cytotoxic effects against human fibrosarcoma and mouse hepatoma cells [115].

### 5.2. Advanced Functional Coating

Beyond polymer coatings, functionalization of MNPs with therapeutic peptides, proteins, and gene vectors offers new possibilities for targeted therapies in NDs.

Glat et al. conjugated iron oxide (γ-Fe_2_O_3_) NPs with a fibrin γ377–395 peptide to extend its half-life and enhance brain delivery. These peptide-conjugated MNPs (21 nm) selectively inhibited microglial activity in tauopathy mouse models, highlighting their potential for tau-targeted neurotherapeutics [116].

In a related approach, Sonawane et al. developed protein-capped metal NPs (PC-NPs)—including PC-Fe_3_O_4_ and PC-CdS—that significantly inhibited tau protein fibrillation in vitro. PC-CdS achieved 63% inhibition and PC-Fe_3_O_4_ 49%. These effects were attributed to surface adsorption, intermediate sequestration, and selective protein–tau interactions. TEM imaging and SDS-PAGE analyses confirmed reduced fibril formation and partial fibril disassembly (up to 88% for PC-CdS at 0.5 mg/mL over 36 h). The protein coating also reduced cytotoxicity, making these systems promising anti-tau candidates, though in vivo studies are still needed [117].

For PD, alpha-synuclein-targeted gene therapy was demonstrated using Fe_3_O_4_ NPs functionalized with a smart hydrogel (N-isopropylacrylamide derivative, NIPAm-AA) responsive to temperature and pH. These NPs delivered shRNA plasmids and nerve growth factor (NGF), reduced alpha-synuclein expression, and protected dopaminergic neurons in MPTP mouse models, improving behavioral outcomes without organ toxicity [30]. Additionally, mesenchymal stem cells labeled with micrometer-sized iron oxide particles were successfully delivered to the brain via intranasal administration in PD models. This non-invasive route bypassed the BBB, resulting in improved motor function and neuronal preservation. Magnetic labeling may enable in vivo tracking, offering a cost-effective and repeatable platform for progressive NDs [118].

**Table 2:** Functionalized MNPs for the treatment of NDs.

Type of Carrier	Coating Agent	Application	Ref.
Polymer Coating			
Protein A conjugated Dextran-coated MNPs	Dextran	Separation of cells, cell membranes, and receptors	[104]
Dextran sulfate-coated SPIONs loaded with Quercetin	Dextran sulfate	Low toxicity in PC12 cells; antioxidant delivery	[105]
Dextran-coated SPIONs	Dextran	Beta-amyloid detection; reduced cytotoxicity in SH-SY5Y cells	[106]
Dextran-coated MNPs	Dextran	Promotion of hMSC migration and dopaminergic neuron regeneration in PD model	[36]
Chitosan-coated MNPs with tacrine	Chitosan	Improved spatial learning and memory in rats; increased Seladin-1 expression	[34]
PEG-coated MNPs	Poly (Ethylene Glycol)	Increased physical stability; increase in blood circulation; targeted delivery	[110]
PEG-coated SPIONs	Poly (Ethylene Glycol)	Enhanced circulation, stability, and targeted delivery	[37]
siRNA-loaded PEGylated SPIONs	Poly (Ethylene Glycol)	BACE1 gene silencing in fibroblast cells; improved cellular uptake	[42]
PEG-block-allyl glycidyl ether (PEG-b-AGE)-SPIONs	Poly (Ethylene Glycol)	Improved sensitivity and specificity for amyloid-beta and tau detection	[111]
Lipid coating			
Congo Red/Rutin-loaded (DSPE)–PEG-coated MNPs	DSPE-PEG-Congo red; DSPE-PEG-phenylboronic acid	Amyloid plaque detection by MRI; targeted delivery of AD therapeutic agents; drug-controlled release by H_2_O_2_ response; prevention of oxidative stress	[113]
Curcumin-loaded DSPE-PEG functionalized with (CRTIGPSVC) and QSH (QSHYRHISPAQV)	DSPE-PEG	Curcumin delivery to the brain; inhibition of amyloid-beta aggregation via NLRP3 suppression	[114]
Advanced functional coating			
Iron oxide (γ-Fe_2_O_3_) nanoparticles conjugated with fibrin γ377–395 peptide	Fibrin γ377–395 peptide	Targeted inhibition of microglial cells in the tauopathy mouse model	[116]
Protein-capped (PC-Fe_3_O_4_/(PC-CdS) metal NPs	Peptides	Inhibition and disassembly of tau fibrils (in vitro)	[117]
Fe_3_O_4_ NPs with NIPAAm derivative	N-isopropylacrylamide derivative	Controlled shRNA release targeting alpha-synuclein; enhanced uptake via NGF receptors	[30]

1,2-dioleoyl-sn-glycero-3-phosphoethanolaminen-[poly(ethylene glycol)]-Congo red (DSPE-PEG-Congo red); 1,2-dioleoyl-sn-glycero3-phosphoethanolamine-n-[poly(ethyleneglycol)]-phenylboronic acid (DSPE-PEG-phenylboronic acid); poly-N-isopropylacrylamide (NIPAAM); nerve growth factor (NGF).

In summary, surface functionalization strategies are critical for translating MNPs into biomedical applications, particularly in NDs. Polymer coatings such as dextran [36,104,119,120], chitosan [33,34,107,108], and PEG [109,110,111] improve stability, prolong circulation, and enable drug and gene delivery, while lipid-based coatings [113,114] and oleic acid [115] enhance membrane interactions and multifunctionality. Advanced functional layers—including peptides, proteins, and gene vectors—add disease specificity and therapeutic activity, exemplified by systems targeting amyloid-β, tau, and α-synuclein [30,116,117]. Each approach offers unique strengths, but also faces limitations such as potential cytotoxicity, stability concerns, or complex synthesis. The most promising directions appear to be PEGylated and lipid-based coatings for multifunctional theranostics, together with peptide- or gene-functionalized systems that directly target pathological proteins in AD and PD. Collectively, these strategies underscore how tailored surface design enables precision delivery and supports the development of clinically relevant MNP-based platforms.

## 6. Biomedical Application of Magnetic Nanoparticles

MNPs have shown substantial potential across a broad spectrum of biomedical applications due to their unique magnetic responsiveness, high biocompatibility, and capacity for surface functionalization. Their versatility supports both in vitro and in vivo uses, ranging from molecular diagnostics to targeted drug delivery.

### 6.1. In Vitro

For decades, polymer-based particles have been used in in vitro diagnostics and biotechnological processes such as immunoassays, nucleic acid separation, and biomolecule immobilization [121]. MNPs offer distinct advantages over conventional polymer particles, particularly their ability to be rapidly and selectively separated from complex biological matrices using external magnetic fields [122].

The first magnetic microspheres with narrow size distribution suitable for biomolecular immobilization were introduced by Ugelstad et al. in 1993 [123]. These MNPs supported various biomolecular attachments, laying the groundwork for advanced magnetic separation technologies. Their high surface-area-to-volume ratio and functionalizable surfaces have since made MNPs indispensable in molecular separation, including the detection and isolation of proteins [124], oligonucleotides, and specific cell types [43].

Magnetic microbeads embedded within polymers are now widely used in cell separation. When mixed with biological samples, they enable the efficient isolation of targeted cells or molecules through magnetic field application. MNPs are also used for real-time detection and visualization of pathogens in rapid diagnostics. For example, Kondo et al. synthesized poly(styrene/N-isopropylacrylamide/methacrylic acid) latex particles embedded with magnetite using a two-step emulsifier-free emulsion polymerization method. Their study showed that increasing magnetite content altered colloidal behavior and thermoflocculation capacity, and prolonged magnetic separation time—demonstrating the influence of magnetic load on particle function [125].

MNPs are further employed in nucleic acid extraction and blood purification. Silica-coated magnetic beads have been successfully used for efficient DNA isolation, as shown by Pinto et al. In therapeutic contexts, MNPs have also been investigated for the removal of inflammatory or autoimmune factors from blood plasma—offering potential treatment avenues for diseases such as sepsis and autoimmune disorders [126].

### 6.2. In Vivo

In in vivo settings, MNPs are highly versatile for both diagnostic and therapeutic purposes. Their large surface area enables conjugation with various biological molecules—including antibodies, peptides, and ligands—facilitating prolonged circulation, specific biomarker recognition, and targeted therapeutic delivery [127]. One of the most well-established diagnostic applications of MNPs is MRI [128]. For such use, MNPs must exhibit low toxicity, excellent biocompatibility, and high colloidal stability in physiological conditions [129]. SPIONs are especially favored, as they do not retain residual magnetization after removal of the external magnetic field, minimizing the risk of particle aggregation and embolism [127,130]. Mouaziz et al. developed submicron amino-dextran-coated magnetic particles and evaluated their efficacy in nucleic acid extraction [119]. Similarly, Yu et al. demonstrated that dextran-coated magnetic fluids were non-toxic and biocompatible, indicating their suitability for systemic administration [120].

Beyond imaging, MNPs are increasingly integrated into magnetically triggered drug delivery systems. These systems enable site-specific, externally controlled drug release via an alternating magnetic field. Early studies by Kost et al. in 1987 demonstrated magnetic regulation of insulin release from ethylene vinyl acetate composites [131]. Later, Paoli et al. showed enhanced dextran release from collagen-based nanocomposites under low-frequency magnetic fields, validating the concept of magnetic field-controlled drug delivery [132].

As the global burden of NDs continues to rise, the demand for effective, targeted, and minimally invasive therapeutic strategies is intensifying. MNPs offer an ideal platform to meet this need by integrating diagnosis, therapy, and targeting into a single nanosystem, paving the way for a new era of personalized medicine and precision neurotherapeutics [127].

Biomedical applications of MNPs clearly illustrate how their physicochemical versatility translates into both diagnostic and therapeutic value. In in vitro systems, functionalized magnetic particles have become indispensable for biomolecule isolation, nucleic acid extraction, and cell separation, where speed, selectivity, and ease of magnetic manipulation confer clear advantages over conventional methods [124,125,126]. In in vivo contexts, applications extend from well-established roles as MRI contrast enhancers to emerging use in magnetically guided and triggered drug delivery [128]. Each of these applications demonstrates unique strengths—such as high sensitivity in imaging or spatial precision in therapy—yet they are also limited by issues including long-term safety, standardization, and scalability. Among the many avenues explored, SPION-based MRI contrast agents and magnetically regulated drug delivery systems stand out as the most clinically advanced and translationally relevant approaches [63,109,129,130]. These examples demonstrate how MNPs bridge laboratory innovation with potential real-world medical impact, setting the stage for continued development toward integrated theranostic platforms in neurodegenerative disease management.

## 7. Applications in Alzheimer’s and Parkinson’s Disease

MNPs represent a rapidly advancing frontier in the diagnosis and treatment of NDs. Their unique physicochemical properties enable multifunctionality, including targeted delivery, molecular imaging, and neuromodulation, across the challenging BBB.

### 7.1. Alzheimer’s Disease

AD is characterized by the accumulation of amyloid-beta plaques and tau tangles, which contribute to synaptic dysfunction and neuronal loss [17]. MNPs have been developed to address both diagnostic and therapeutic challenges associated with this pathology [127]. SPIONs functionalized with amyloid-beta oligomer-specific single-chain variable fragment antibodies and class A scavenger receptor activators have shown dual functionality: targeting toxic amyloid-beta species and promoting microglial clearance, while also serving as MRI contrast agents for non-invasive monitoring [133]. A promising non-invasive therapeutic strategy involves SPION-loaded, chitosan-coated bilosomes designed for magnetic nose-to-brain delivery. In this approach, resveratrol—a potent antioxidant—was encapsulated alongside SPIONs to enable magnetic targeting to the olfactory region using an external magnetic field. The chitosan coating improved mucosal adhesion and residence time, while the magnetic responsiveness ensured localized delivery past the nasal barrier. This platform bypassed the BBB, increased brain bioavailability of resveratrol, and minimized systemic exposure, offering a patient-friendly route for long-term AD therapy that could be extended to other NDs [35]. Another promising avenue for targeted drug delivery in AD involves transferrin-functionalized magnetic nanoformulations. These NPs exploit transferrin receptors, which are highly expressed on brain capillary endothelial cells, to cross the BBB via receptor-mediated transcytosis. In a study conducted by Cui et al., MNPs were encapsulated in a PLGA-PEG copolymer and surface-modified with anti-transferrin monoclonal antibody (OX26), forming NPs with controlled size, good stability, and strong magnetic properties. In vitro evaluations confirmed their potential for brain-specific delivery of neurotherapeutics [134]. In a related strategy, curcumin—a natural polyphenol with strong affinity for amyloid-beta—was conjugated to SPIONs to develop a dual-function system for both targeting and detecting amyloid plaques. These curcumin-functionalized MNPs maintained superparamagnetic behavior and exhibited strong binding to amyloid-beta aggregates in vitro. Notably, the conjugates enhanced T_2_-weighted MRI contrast and demonstrated significant potential for non-invasive plaque detection, eliminating the need for antibodies or radiolabels. This system combines biocompatibility, amyloid specificity, and magnetic signal enhancement, providing a simplified, ligand-based diagnostic platform for early AD imaging and potentially targeted intervention [135]. Recently, a highly sensitive nanotheranostic platform was developed by conjugating curcumin with SPIONs coated with DSPE-PEG and functionalized with CRT and QSH peptides. This system effectively crossed the BBB, targeted early amyloid-beta plaques, and allowed for 3D MRI-based quantification of plaque volume. Moreover, it reversed memory deficits in APP/PS1 mice, likely through inhibition of the NLRP3 inflammasome, and enhanced brain-derived neurotrophic factor-mediated neurogenesis without the need for vascular permeability enhancers. This system combines biocompatibility, amyloid specificity, anti-inflammatory effects, and magnetic signal enhancement, offering a multifunctional approach for early AD diagnosis and treatment [114].

### 7.2. Parkinson’s Disease

In PD, where dopaminergic neuron loss and alpha-synuclein aggregation underlie progressive motor dysfunction, MNPs offer both diagnostic and therapeutic potential [6,40]. MNPs functionalized with alpha-synuclein-specific peptides have been developed to serve as MRI contrast agents capable of detecting early-stage protein aggregation, enabling non-invasive disease monitoring [26]. Therapeutically, MNPs are being used to enhance the precision and efficacy of regenerative approaches. For instance, human adipose-derived stem cells labeled with MNPs can be guided magnetically to lesioned brain regions. In murine PD models, this targeted delivery preserved dopaminergic neurons and improved behavioral outcomes [136]. In addition, magnetothermal neuromodulation has emerged as a non-invasive alternative to deep brain stimulation. In this approach, MNPs are injected into specific brain regions and remotely activated by alternating magnetic fields, leading to localized heating that modulates neuronal activity. This strategy has successfully alleviated motor symptoms in PD models without the need for implanted electrodes [137].

Recent studies have also explored the physical guidance of neuronal growth using MNPs. Neurons or neural progenitors internalized with MNPs can be manipulated using magnetic field gradients to direct neurite extension toward target regions, potentially enabling reconstruction of damaged dopaminergic pathways [138]. An advanced variation utilizes streptavidin-biotin-modified magnetic actuators anchored to the neuronal membrane to apply remote mechanical forces. When stimulated by an external magnetic field, these NPs guide the direction of cellular growth in vitro by exerting precise tension on developing neurites [139]. Another work also demonstrates the feasibility of in vivo MRI tracking of SPION-labeled olfactory ensheathing cells transplanted into spinal cord tissues. These cells, known to facilitate axonal regeneration and remyelination, were tracked for up to 2 months post-grafting, showing clear MRI signal correlation with histological markers such as p75 and Prussian blue stain [140].

Innovative work has also explored dual-stimuli responsive materials incorporating PD-relevant proteins and multifunctional NPs. A nanostructured film composed of MNPs, gold NPs, and alpha-synuclein was fabricated to investigate material self-assembly and responsiveness to both magnetic and thermal stimuli. The alpha-synuclein served as a self-assembling matrix protein, while embedded NPs provided physical responsiveness. This platform holds promises for future applications in biosensing, localized actuation, and stimuli-responsive drug delivery, particularly where alpha-synuclein pathology intersects with targeted neurotherapeutic interventions. Such smart materials may be further engineered for PD-relevant diagnostic or therapeutic systems [141]. Another promising nanocarrier design involves the use of magnetoliposomes, which integrate superparamagnetic NPs within lipid bilayers to enable both controlled drug release and magnetic targeting. These multifunctional carriers exhibited high drug loading efficiency, colloidal stability, and responsiveness to external magnetic fields [142].

MNP-based strategies in AD and PD demonstrate clear potential by enabling early biomarker detection, targeted delivery, and functional modulation across the BBB [114,137]. While antibody- and ligand-functionalized systems offer high specificity, challenges remain in stability, reproducibility, and translation [26,35,133,134,136]. The most promising directions are SPION-based imaging probes, magnetically guided regenerative approaches, and non-invasive neuromodulation, which together highlight MNPs as versatile theranostic tools for neurodegenerative disease management [37].

## 8. Challenges and Future Perspectives

While MNPs show great promise for the diagnosis and treatment of NDs, several critical challenges must be addressed before they can be widely implemented in clinical settings. These challenges pertain to their biocompatibility, long-term safety, scalability, and regulatory acceptance.

One of the foremost concerns is the potential toxicity and immunogenicity of MNPs. Although many MNPs, especially those coated with biocompatible polymers such as dextran, PEG, or chitosan, exhibit low acute toxicity, their long-term effects in vivo remain poorly understood. The accumulation of iron-based NPs in organs such as the liver, spleen, or brain can induce oxidative stress, inflammatory responses, or interfere with normal cellular function [38,143]. Therefore, extensive toxicological studies are necessary to evaluate the degradation, biodistribution, and clearance of NPs from the body. Factors such as particle size, surface charge, and coating stability greatly influence these outcomes and must be carefully optimized during the design process.

In addition to safety, the reproducible and cost-effective synthesis of MNPs with uniform size, shape, and surface chemistry remains a manufacturing challenge. Achieving batch-to-batch consistency is essential for clinical translation and regulatory approval. Current synthesis methods must be adapted to comply with GMP standards, and robust quality control measures need to be established to ensure purity, stability, and performance reproducibility [67].

Another hurdle is the regulatory pathway for NP-based diagnostics and therapeutics. Regulatory bodies, such as the FDA and EMA, require a detailed characterization of the physicochemical properties, pharmacokinetics, toxicity, and therapeutic efficacy of these materials. However, existing regulatory frameworks are not always well-suited for multifunctional or theranostic systems that integrate both imaging and therapy. This necessitates the development of new guidelines tailored to nanomedicine and interdisciplinary collaboration between scientists, clinicians, and policymakers.

Innovations such as magnetogenetics—where magnetic fields are used to control genetically engineered cellular functions—and integration with artificial intelligence for data-driven optimization of nanocarrier design are also gaining attention [66,144]. Moreover, coupling MNPs with wearable or implantable biosensors could facilitate continuous, non-invasive monitoring of ND biomarkers in real-time, revolutionizing long-term patient care and disease management.

Looking forward, further progress will require not only overcoming technical and regulatory hurdles but also ensuring scalability through environmentally sustainable synthesis methods. In parallel, the establishment of standardized protocols for evaluating safety, biodistribution, and therapeutic efficacy will be critical to harmonize research outcomes across laboratories. These efforts will ultimately determine whether functionalized MNPs can be reliably advanced from experimental platforms to routine clinical use.

## 9. Materials and Methods

A structured literature search was performed in PubMed, Scopus, Web of Science, and Google Scholar. Search terms included: magnetic nanoparticles, iron oxide nanoparticles, functionalization, surface modification, Alzheimer’s disease, Parkinson’s disease, neurodegenerative disorders and drug delivery. Eligible studies were peer-reviewed articles in English that focused on synthesis methods, surface functionalization, or biomedical applications of magnetic nanoparticles relevant to neurodegenerative disorders. Exclusion criteria were non-English texts, conference abstracts, and studies without clear relevance to the reviewed area. After removing duplicates, full texts were reviewed for eligibility, and reference lists were screened to capture additional relevant publications.

## 10. Conclusions

Functionalized magnetic nanoparticles, especially those based on iron oxide, show significant promise as innovative tools for managing neurodegenerative disorders. Based on current evidence, we can view their potential optimistically. Although they may not yet fully “revolutionize” treatment, functionalized MNPs offer transformative possibilities by combining diagnosis and therapy into a single platform. Their ability to cross the blood–brain barrier, act as MRI contrast agents, facilitate targeted drug and gene delivery, and interact with disease-specific proteins such as amyloid-β, tau, and α-synuclein positions them as strong candidates for precision neurotherapeutics. However, several challenges still exist regarding long-term safety, large-scale reproducibility, and regulatory approval. Key research findings indicate that polymer- and lipid-functionalized MNPs have shown improved stability, circulation, and targeting. Additionally, advanced peptide- and gene-functionalized systems provide disease specificity, while superparamagnetic iron oxide nanoparticle (SPION)-based models and magnetically guided delivery strategies represent some of the most clinically relevant applications. Overall, these advancements suggest that functionalized MNPs are on a promising trajectory to transform the diagnosis and treatment of Alzheimer’s and Parkinson’s diseases, provided that future research successfully addresses the remaining translational challenges.

## Data Availability

No new data were created or analyzed in this study. Data sharing is not applicable to this article.

## Abbreviations

The following abbreviations are used in this manuscript:

AD	Alzheimer’s disease
BBB	Blood–brain barrier
CNS	Central nervous system
DSPE-PEG	1,2-dioleoyl-sn-glycero-3-phosphoethanolaminen-[poly(ethylene glycol)]
EVs	Extracellular vesicles
IONPs	Iron oxide nanoparticles
MNPs	Magnetic nanoparticles
MRI	Magnetic resonance imaging
NDs	Neurodegenerative disorders
NGF	Nerve growth factor
NIPAAM	poly-N-isopropylacrylamide
NPs	Nanoparticles
PC-CdS	Protein-capped cadmium sulfide
PC-Fe_3_O_4_	Protein-capped Fe_3_O_4_
PC-NPs	Protein-capped nanoparticles
PD	Parkinson’s disease
PEG	Polyetylenglycol
PLA	Polylactic acid
RNS	Reactive nitrogen species
ROS	Reactive oxygen species
SPION	Superparamagnetic iron oxide nanoparticles
TEM	Transmission electron microscopy
